# Preoperatively predicting the lymph node metastasis and prognosis for gastric cancer patients

**DOI:** 10.1038/s41598-024-61671-6

**Published:** 2024-05-16

**Authors:** Danfang Wang, Yaxin Wang, Lin Dong, Xin Zhang, Jianfei Du

**Affiliations:** 1 Department of Oncology, Xi’an Gaoxin Hospital, 16 Tuanjie South Road, Xi’an, 710075 Shaanxi People’s Republic of China; 2 Department of Oncology Surgery, First Affiliated Hospital of Xi’an Jiaotong University, Xi’an, China

**Keywords:** Cancer screening, Biomarkers, Gastroenterology

## Abstract

The preoperative distinguishment of lymph nodes (LN) with metastasis plays a pivotal role in guiding the surgical extension for gastric cancer (GC). We aim to identify the preparative risk factors for LN metastasis in GC patients. We retrospectively reviewed 424 patients who underwent radical GC resection in our medical center between Jan 2011 and Dec 2018. Multivariate logistic regression was employed to identify risk factors for LN metastasis, while multivariate COX regression was utilized to evaluate prognostic factors. The median overall survival of patients with or without LN metastases was 31 and 58 months, respectively. In multivariate analysis, lower albumin (OR = 0.512; P = 0.004) and prealbumin (OR = 0.367, P = 0.001) and higher CEA (OR = 3.178, P < 0.001), CA199 (OR = 2.278, P = 0.002) and platelets (OR = 1.697, P = 0.017) were found to be significantly associated with LN metastasis. In survival analysis, older age (HR = 1.712), larger tumors (HR = 1.082), higher D-dimer (HR = 1.561) and CA199 (HR = 1.553), advanced staging (stage II, HR = 3.446; stage III-IV, HR = 11.089), lower prealbumin levels (lower level for reference, HR = 0.63), and absence of adjuvant chemotherapy (HR = 0.396) was discovered to be associated with poorer overall survival (all P < 0.05). In conclusion, our results demonstrated that preoperative prealbumin-bound tumor markers can effectively predict LN metastasis. Additionally, prealbumin was found to possess prognostic value as well.

## Introduction

Although global morbidity and mortality have declined over the past few decades, gastric cancer (GC) remains the fifth most commonly diagnosed disease and the fourth leading cause of cancer-related death^1^. 679,100 new cases of gastric cancer are diagnosed in China every year^2^.

The decision to require perioperative chemotherapy is primarily based on TNM staging. Given that preoperative clinical TNM staging is primarily determined by imaging methods with limited sensitivity^3^, it would be beneficial to use available laboratory biomarkers to assess preoperative LN involvement. Malnutrition is common in cancer patients and is associated with a patient’s response to chemotherapy. Nutritional assessment in patients with cancer may improve patient quality of life^4^. As a nutritional marker, albumin has been reported to be implicated in lymph node metastasis^5,6^. Prealbumin is the earliest laboratory indicator of nutritional status and has emerged as the preferred marker for malnutrition^7^. However, evidence regarding the relationship of prealbumin and LN metastasis is very limited. Our study retrospectively sought to evaluate the predictive role of common blood markers especially albumin and prealbumin in lymph node metastasis and their respective prognostic value.

## Results

### Clinicopathological features of patients

A total of 424 patients were enrolled in the present study, of which 311 (73.3%) were males and 113 (26.7%) were females. The number of patients with LN negative and LN positive (N1, 71; N2, 69; N3, 105) was 179 and 245, respectively. Patient characteristics, grouped according to LN metastasis status, are presented in Table 1. It was found that LN metastasis occurred more frequently in patients with larger tumor size (P < 0.001), advanced TNM stage (P < 0.001), poor differentiation (P = 0.008), positive cancer embolus (P < 0.001), and total GC distribution (P < 0.001). Furthermore, patients with LN metastasis demonstrated significantly higher levels of FIB (P = 0.015), D-dimer (P = 0.001), FDP (P < 0.001), CEA (P < 0.001), CA199 (P = 0.02), and platelets (P = 0.049), as well as significantly lower levels of albumin (P = 0.001), prealbumin (P = 0.001), and hemoglobin (P = 0.03) (Table 1).

**Table 1:** Demographic and baseline characteristics of patients grouped by lymph node (LN) status.

Characteristic	Overall (N = 424)	LN negative (N = 179)	LN positive (N = 245)	P value
Gender				0.948
Male	311 (73.3)	131 (73.2)	180 (73.5)	
Female	113 (26.7)	48 (26.8)	65 (26.5)	
Age, years				0.318
< 60	192 (45.3)	76 (42.5)	116 (47.3)	
≥ 60	232 (54.7)	103 (57.5)	129 (52.7)	
Tumor size, cm	4 (2.5–6)	3 (2–4.5)	4.5 (3–6.63)	< 0.001
pT				< 0.001
1	92 (21.7)	74 (41.3)	18 (7.3)	
2	36 (8.5)	24 (13.4)	12 (4.9)	
3	45 (10.6)	21 (11.7)	24 (9.8)	
4	251 (59.2)	60 (33.5)	191 (78)	
TNM stage				< 0.001
I	108 (25.5)	96 (53.6)	12 (4.9)	
II	57 (13.4)	34 (19)	23 (9.4)	
III-IV	259 (61)	49 (27.4)	195 (79.6)	
Adjuvant chemotherapy				< 0.001
No	171 (40.3)	111 (62)	60 (24.5)	
Yes	253 (59.7)	68 (38)	185 (75.5)	
Differentiation				0.008
Poorly	297 (70)	113 (63.1)	184 (75.1)	
Moderately-Well	127 (30)	66 (36.9)	61 (24.9)	
Cancer embolus				< 0.001
No	350 (82.5)	175 (97.8)	175 (71.4)	
Yes	74 (17.5)	4 (2.2)	70 (28.6)	
Tumor location				< 0.001
Proximal	111 (26.2)	102 (57)	124 (50.6)	
Distal	226(53.3)	56 (31.3)	55 (22.4)	
Total	87 (20.5)	21 (11.7)	66 (26.9)	
Albumin, g/L	38.8 (35.6–41.8)	39.8 (36.5–42.6)	38.41(34.9–40.7)	0.001
Prealbumin, mg/L	208 ± 63.8	219.8 ± 60	199.3 ± 65.29	0.001
Fibrinogen, g/L	3.2 (2.65–3.88)	3.1 (2.5–3.65)	3.23 (2.71–4.09)	0.015
D-dimer, mg/L	0.5 (0.1–0.8)	0.4 (0–0.6)	0.5 (0.2–0.96)	0.001
FDP, mg/L	1 (0.6–1.7)	0.9 (0.5–1.5)	1.1 (0.7–1.9)	< 0.001
CEA, ng/mL	2.295 (1.33–4.36)	2.09 (1.21–3.1)	2.57 (1.43–6.51)	< 0.001
CA199, U/mL	10.24 (6.16–20.06)	9.56 (5.98–16.05)	11.5 (6.29–27.26)	0.02
WBC, 10^9/L	5.41 (4.26–6.62)	5.11 (4.15–6.58)	5.5 (4.4–6.66)	0.197
Hb, g/L	129 (108.25–143)	132 (113.5–144)	127 (105–142)	0.03
Neutrophil, 10^9/L	3.09 (2.33–4.19)	2.98 (2.28–4.15)	3.26 (2.43–4.21)	0.112
Lymphocyte, 10^9/L	1.56 (1.24–1.92)	1.58 (1.26–1.92)	1.54 (1.21–1.92)	0.297
Monocyte, 10^9/L	0.385 (0.27–0.5)	0.36 (0.27–0.52)	0.4 (0.29–0.49)	0.382
Platelet, 10^9/L	205 (160–255)	194.5 (154.5–248.25)	212 (166.5–257)	0.049
OS	38 (22.25–62)	58 (34–67)	31 (14–44)	< 0.001
DFS	38 (20.25–62)	58 (34–67)	30 (13–44)	< 0.001

Values in parentheses are percent or interquartile ranges.

*FDP* fibrin degradation products, *CEA* carcinoembryonic antigen, *CA199* carbohydrate antigen 199, *WBC* white blood cell, *Hb* hemoglobin, *OS* overall survival, *DFS* disease free survival.

### Risk factors for LN metastasis

The cutoff values for CEA, CA199, albumin, prealbumin, FIB, D-dimer, FDP, hemoglobin, platelets, leukocytes, lymphocytes, monocytes, and neutrophils were 4.42 ng/mL, 19.07 U/mL, 39.75 g/L, 159.1 mg/L, 3.78 g/L, 0.965 mg/L, 0.65 mg/L, 113.5 g/L, 188.5 10^9/L, 4.915 10^9/L, 1.745 10^9/L, 0.366 10^9/L, and 3.12 10^9/L, respectively.

Univariate logistic regression analysis showed that lower levels of albumin, prealbumin, and hemoglobin, as well as higher levels of FIB, D-dimer, CEA, CA199, white blood cell (WBC) count, neutrophils, monocytes, and platelets, were identified as risk factors for LN metastasis.

In multivariate logistic regression analysis, higher levels of CEA (odds ratio [OR]: 3.718, 95% confidence interval [CI]: 2.077–6.657), CA199 (OR: 2.278, 95% CI: 1.352–3.838), and platelets (OR: 1.697, 95% CI: 1.098–2.624) were found to be independent risk factors for LN metastasis. Conversely, higher levels of albumin (OR: 0.512, 95% CI: 0.327–0.803) and prealbumin (OR: 0.367, 95% CI: 0.202–0.667) were associated with a lower risk of LN metastasis (Table 2). Using the N0 stage as a control, we performed multiple logistic regression analysis of the above independent risk factors. It was found that lower levels of prealbumin were associated with an increased risk of N1 (OR: 2.674, 95% CI: 1.273–5.617), N2 (OR: 2.852, 95% CI: 1.341–6.064), and N3 (OR: 2.675, 95% CI: 1.342–5.336) stage compared with elevated levels, while platelets were not significantly associated with any three stages (Table 3).

**Table 2:** Univariate and multivariate analyses of variables for lymph node metastasis.

	Univariate analysis			Multivariate analysis		
	Odds ratio	95% CI	P value	Odds ratio	95% CI	P value
Gender	1.015	0.656–1.569	0.948			
Age	0.821	0.557–1.21	0.318			
Albumin	0.451	0.302–0.675	< 0.001	0.512	0.327–0.803	0.004
Prealbumin	0.258	0.148–0.452	< 0.001	0.367	0.202–0.667	0.001
Fibrinogen	1.936	1.241–3.021	0.004			0.222
D-dimer	2.636	1.524–4.558	0.001			0.176
FDP	1.97	1.284–3.021	0.002			0.071
CEA	4.667	2.682–8.121	< 0.001	3.718	2.077–6.657	< 0.001
CA199	2.555	1.583–4.122	< 0.001	2.278	1.352–3.838	0.002
WBC	1.641	1.105–2.438	0.014			0.22
Hb	0.626	0.407–0.964	0.033			0.73
Neutrophil	1.644	1.112–2.431	0.013			0.267
Lymphocyte	0.679	0.453–1.019	0.061			
Monocyte	1.522	1.013–2.25	0.035			0.927
Platelet	1.726	1.161–2.566	0.007	1.697	1.098–2.624	0.017

The optimal cut-off value was 39.75 g/L for albumin, 159.1 mg/L for prealbumin, 3.78 g/L for fibrinogen, 0.965 mg/L for D-dimer, 0.65 mg/L for FDP, 4.42 ng/mL for CEA, 19.07 U/mL for CA199, 4.915 *10^9/L for WBC, 113.5 *10^9/L for Hb, 3.12 *10^9/L for neutrophil, 1.745 *10^9/L for lymphocyte, 0.366 *10^9/L for monocyte and 188.5 *10^9/L for platelet. The reference for gender, age and peripheral blood indexes were female, age < 60 years and the lower level of peripheral blood indexes according to their cutoff values.

*CI* confidence interval.

**Table 3:** Multivariate multiple logistic regression analyses of variables for LN metastasis.

	N1 stage			N2 stage			N3 stage		
	Odds ratio	95% CI	P value	Odds ratio	95% CI	P value	Odds ratio	95% CI	P value
Albumin	2.065	1.099–3.879	0.024	1.708	0.905–3.222	0.098	2.12	1.199–3.749	0.01
Prealbumin	2.674	1.273–5.617	0.009	2.852	1.341–6.064	0.006	2.675	1.342–5.336	0.005
CEA	0.449	0.209–0.967	0.041	0.243	0.118–0.498	< 0.001	0.207	0.108–0.397	< 0.001
CA199	0.533	0.268–1.06	0.073	0.386	0.198–0.754	0.005	0.407	0.221–0.751	0.004
Platelet	0.552	0.303–1.006	0.052	0.635	0.345–1.167	0.143	0.595	0.346–1.023	0.06

For multiple logistic regression analyses the reference category is N0 stage. The reference of parameters was albumin ≥ 39.75, prealbumin ≥ 159.1, CEA ≥ 4.42, CA199 ≥ 19.07 and platelet ≥ 188.5.

**Figure 1:**
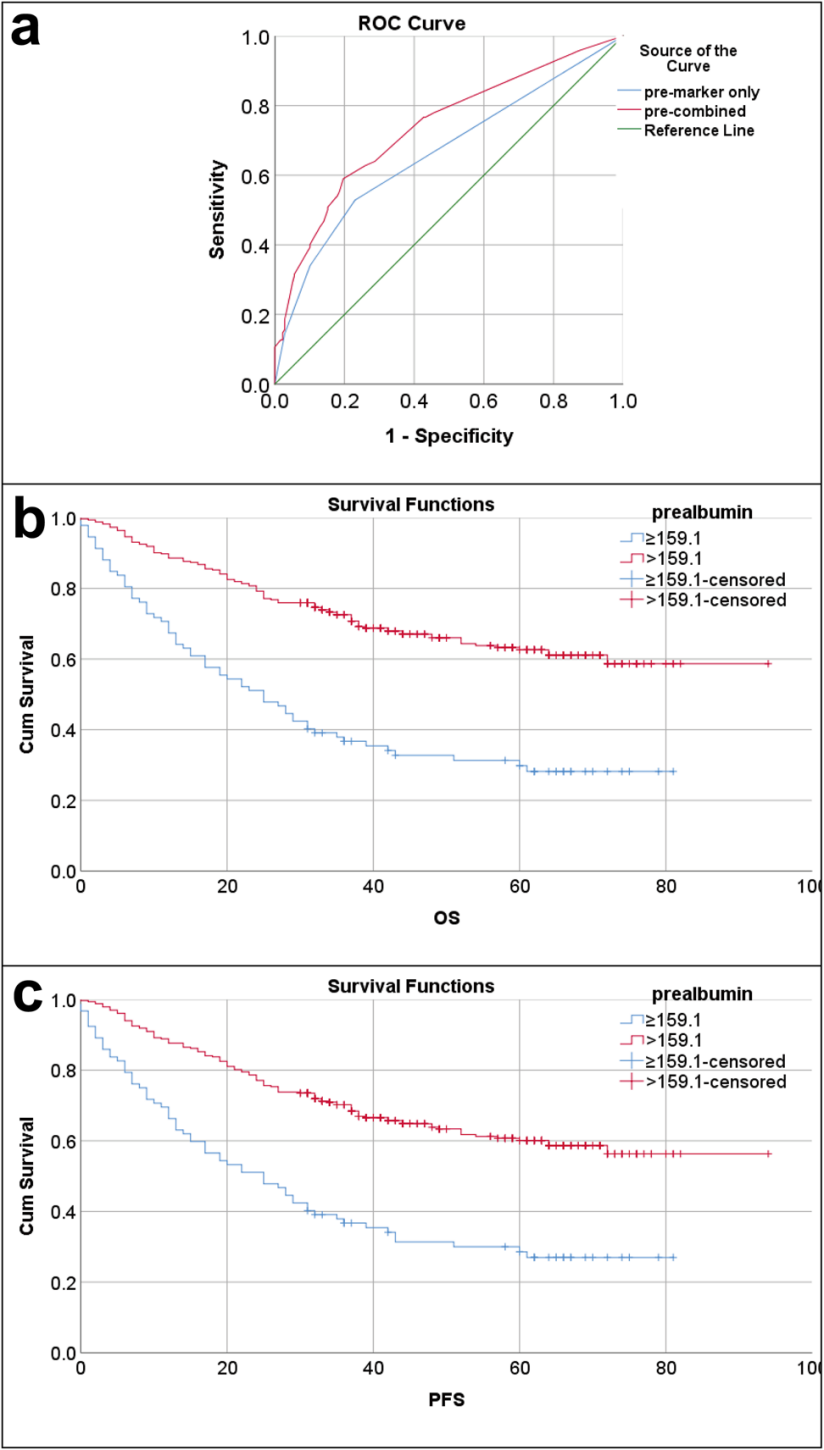
ROC curves and Kaplan–Meier survival curves. (**a**) ROC curve analysis for predicting lymph node metastasis. Pre-markers only represent ROC curve combining CEA and CA199; pre-combined represent ROC curve combining CEA, CA199, albumin, prealbumin and platelet. (**b**) Kaplan–Meier survival curves of prealbumin for overall survival (OS); the 5 year survival rate of low prealbumin group was 29.8%, of high prealbumin group was 62.6%. (**c**) Kaplan–Meier survival curves of prealbumin for disease-free survival (DFS); the 5 year survival rate of low prealbumin group was 28.6%, of high prealbumin group was 60.1%.

To evaluate lymph node metastasis more efficiently, we conducted a combined ROC curve analysis. The analysis, which combined CEA and CA199 (referred to as Combined P1), yielded an area under the curve (AUC) of 0.664, a sensitivity of 0.531, and a specificity of 0.765. Additionally, the ROC analysis that combined CEA, CA199, albumin, prealbumin, and platelets (referred to as Combined P2), indicated an AUC of 0.736, a sensitivity of 0.59, and a specificity of 0.803 (Fig. 1a). Importantly, according to Table 4, the diagnostic efficacy of Combined P2 was significantly higher than that of Combined P1 (P < 0.001).

**Table 4:** The diagnostic efficacy of indicators for lymph node metastasis.

	AUC	95% CI	Cut-off	Sen	Spe	Youden index	PPV	NPV	P value
CEA	0.608	0.555–0.661	4.42	0.343	0.899	0.242	0.824	0.500	0.008
CA199	0.566	0.512–0.62	19.07	0.331	0.838	0.169	0.736	0.478	< 0.001
Albumin	0.597	0.542–0.652	39.75	0.508	0.678	0.186	0.455	0.351	0.057
Prealbumin	0.594	0.54–0.648	159.1	0.899	0.302	0.201	0.515	0.196	0.04
Platelet	0.556	0.5–0.612	188.5	0.664	0.466	0.13	0.630	0.503	0.003
Combined P1	0.664	0.613–0.715	0.554	0.531	0.765	0.296	0.756	0.544	Reference
Combined P2	0.736	0.689–0.783	0.591	0.59	0.803	0.393	0.804	0.588	< 0.001

*AUC* area under receiver operating characteristics, *CI* confidence interval, *Sen* sensitivity, *Spe* specificity, *PPV* positive predictive value, *NPV* negative predictive value.

P, P value for comparison of AUC of reference with other indicators using DeLong’s test; Combined P1, ROC analysis combining CEA and CA199; Combined P2, ROC analysis combining CEA, CA199, albumin, prealbumin and platelet.

### Independent prognostic factor for OS and DFS

The Kaplan–Meier survival curves revealed that patients with high prealbumin had significantly longer overall survival (OS) and disease-free survival (DFS) compared to those with low prealbumin levels (Fig. 1b-c). Furthermore, the univariate analysis demonstrated that older age, larger tumor size, TNM stage II and TNM stage III, positive tumor thrombus, proximal and total gastric cancer, higher levels of fibrinogen, D-dimer, FDP, CEA, CA199, neutrophils, and monocytes were identified as poor prognostic factors. Conversely, higher levels of albumin, prealbumin, hemoglobin, and the receipt of adjuvant chemotherapy were associated with prolonged survival.

Multivariate COX regression analysis identified age ≥ 60 years as a significant risk factor for overall survival (OS) (Hazard ratio (HR): 1.712, 95% confidence interval (CI): 1.197–2.4, P = 0.003). Additionally, larger tumor size (HR: 1.082, 95% CI: 1.023–1.1, P = 0.006), advanced TNM stage (TNM stage II, HR: 3.446, 95% CI: 1.323–8.9, P = 0.011; TNM stage III-IV, HR: 11.089, 95% CI: 5.039–24.405, P < 0.001), total gastric cancer (GC) (HR: 1.695, 95% CI: 1.086–2.644, P = 0.02), higher D-dimer (HR: 1.561, 95% CI: 1.071–2.276, P = 0.021), and higher CA199 (HR: 1.553, 95% CI: 1.09–2.214, P = 0.015) were also identified as independent risk factors for OS. Conversely, higher levels of prealbumin (HR: 0.63, 95% CI: 0.433–0.918, P = 0.016) and utilization of adjuvant chemotherapy (HR: 0.396, 95% CI: 0.26–0.603, P < 0.001) were found to be independent protective factors (Table 5). Moreover, we further confirmed the independent prognostic effect of prealbumin on disease-free survival (DFS) (HR: 0.653, 95% CI: 0.452–0.944, P = 0.023), as well as age, tumor size, TNM stage, tumor location, D-dimer, and CEA (Table 6).

**Table 5:** Univariate and multivariate analyses for overall survival of GC patients.

Parameters	Univariate analysis			Multivariate analysis		
	HR	95% CI	P value	HR	95% CI	P value
Gender	1.137	0.811–1.594	0.456			
Age, years	1.623	1.198–2.199	0.002	1.712	1.197–2.447	0.003
Tumor size	1.14	1.095–1.187	< 0.001	1.082	1.023–1.144	0.006
TNM stage						
I	1			1		
II	1.771	0.781–4.013	0.171	3.446	1.323–8.974	0.011
III-IV	8.095	4.489–14.598	< 0.001	11.089	5.039–24.405	< 0.001
Adjuvant chemotherapy	1.669	1.21–2.303	0.002	0.396	0.26–0.603	< 0.001
Differentiation	0.748	0.537–1.041	0.085			
Cancer embolus	1.864	1.312–2.648	0.001			0.303
Tumor location						
Distal	1			1		
Proximal	0.671	0.466–0.964	0.031	0.917	0.603–1.395	0.685
Total	2.281	1.552–3.352	< 0.001	1.695	1.086–2.644	0.02
Albumin	0.503	0.36–0.704	< 0.001			0.688
Prealbumin	0.357	0.263–0.485	< 0.001	0.63	0.433–0.918	0.016
Fibrinogen	2.104	1.563–2.833	< 0.001			0.221
D-dimer	2.529	1.836–3.484	< 0.001	1.561	1.071–2.276	0.021
FDP	1.767	1.234–2.53	0.002			0.738
CEA	2.061	1.51–2.814	< 0.001			0.122
CA199	1.848	1.362–2.507	< 0.001	1.553	1.09–2.214	0.015
WBC	1.276	0.938–1.735	0.12			
Hb	0.657	0.484–0.891	0.007			0.452
Neutrophil	1.422	1.058–1.912	0.02			0.142
Lymphocyte	0.748	0.544–1.028	0.074			
Monocyte	1.563	1.147–2.128	0.005			0.224
Platelet	1.075	0.794–1.455	0.642			

The cut-off value was 39.75 g/L for albumin, 159.1 mg/L for prealbumin, 3.78 g/L for fibrinogen, 0.965 mg/L for D-dimer, 0.65 mg/L for FDP, 4.42 ng/mL for CEA, 19.07 U/mL for CA199, 4.915 *10^9/L for WBC, 113.5 *10^9/L for Hb, 3.12 *10^9/L for neutrophil, 1.745 *10^9/L for lymphocyte, 0.366 *10^9/L for monocyte and 188.5 *10^9/L for platelet. The reference of gender, age, adjuvant chemotherapy, differentiation and cancer embolus were female, < 60 years, without chemotherapy, poorly differentiation and no cancer embolus. All peripheral blood indexes were divided into two groups according to the cutoff value, and low levels were used as the control group for survival analysis.

*HR* hazard ratio, *CI* confidence interval.

**Table 6:** Univariate and multivariate analyses for progression-free survival of GC patients.

Parameters	Univariate analysis			Multivariate analysis		
	HR	95% CI	P value	HR	95% CI	P value
Gender	1.125	0.811–1.561	0.481			
Age	1.538	1.146–2.063	0.004	1.495	1.064–2.101	0.021
Tumor size	1.141	1.097–1.186	< 0.001	1.06	1.002–1.12	0.041
TNM stage						
I	1			1		
II	1.957	0.907–4.223	0.087	3.716	1.516–9.107	0.004
III-IV	7.925	4.494–13.974	< 0.001	10.024	4.706–21.353	< 0.001
Adjuvant chemotherapy	1.677	1.226–2.293	0.001	0.416	0.279–0.62	< 0.001
Differentiation	0.754	0.547–1.04	0.085			
Cancer embolus	2	1.428–2.802	< 0.001			0.662
Tumor location						
Distal	1			1		
Proximal	0.681	0.478–0.971	0.034	0.957	0.634–1.445	0.836
Total	2.361	1.621–3.44	< 0.001	1.761	1.145–2.709	0.01
Albumin	0.494	0.356–0.685	< 0.001			0.4
Prealbumin	0.38	0.281–0.513	< 0.001	0.653	0.452–0.944	0.023
Fibrinogen	1.978	1.479–2.645	< 0.001			0.365
D-dimer	2.464	1.801–3.37	< 0.001	1.536	1.067–2.213	0.021
FDP	1.796	1.266–2.548	0.001			0.714
CEA	2.186	1.618–2.955	< 0.001	1.554	1.095–2.205	0.013
CA199	1.744	1.293–2.363	< 0.001			0.081
WBC	1.242	0.922–1.672	0.154			
Hb	0.653	0.486–0.879	0.005			0.64
Neutrophil	1.421	1.066–1.895	0.017			0.117
Lymphocyte	0.737	0.541–1.004	0.053			
Monocyte	1.401	1.042–1.885	0.025			0.672
Platelet	1.059	0.789–1.423	0.701			

HR, hazard ratio; CI, confidence interval. The cut-off value was 39.75 g/L for albumin, 159.1 mg/L for prealbumin, 3.78 g/L for fibrinogen, 0.965 mg/L for D-dimer, 0.65 mg/L for FDP, 4.42 ng/mL for CEA, 19.07 U/mL for CA199, 4.915 *10^9/L for WBC, 113.5 *10^9/L for Hb, 3.12 *10^9/L for neutrophil, 1.745 *10^9/L for lymphocyte, 0.366 *10^9/L for monocyte and 188.5 *10^9/L for platelet. The reference of gender, age, adjuvant chemotherapy, differentiation and cancer embolus were female, < 60 years, without chemotherapy, poorly differentiation and no cancer embolus. All peripheral blood indexes were divided into two groups according to the cutoff value, and low levels were used as the control group for survival analysis.

## Discussion

Our current study demonstrates the significance of albumin, prealbumin and platelets in preoperative peripheral blood for LN metastasis in GC. The ROC curve indicated that the utility of albumin, prealbumin and platelets as predictive markers for LN metastasis in GC was comparable to that of traditional GC markers, namely CEA and CA199. The combined application of these indicators improves the efficacy for predicting LN metastasis. Prealbumin was also an independent factor for patient survival in all analyzed blood parameters, as were as D-dimer and GC markers.

Neoadjuvant chemotherapy has shown a survival benefit relative to surgery alone^8^, and the possibility of LN metastasis is a key consideration for such treatment. Neoadjuvant chemotherapy is recommended for patients with lymph node metastases in Japan^9^. It has been reported that neoadjuvant chemotherapy can effectively control LN metastasis in GC, thereby reducing tumor N stage and increasing complete resection^10^. Therefore, preoperative assessment of LN status provides a reference for choosing a reasonable treatment plan for patients.

Tumor markers are commonly used to predict prognosis, monitor a positive course, and detect recurrence^11,12^. As traditional tumor markers, CEA and CA199 may help detect positive lymph nodes^13,14^. In our current study, we assessed the predictive value of preoperative peripheral blood-derived clinical routine laboratory biomarkers for LN metastasis. Our data showed that the LN metastasis group had significantly elevated CEA and CA199. Multivariate regression analysis showed their clinical significance for predicting LN metastasis. Previous studies reported that CEA, CA199, CA724 and CA242 were all associated with LN metastasis, and their combination could improve diagnostic efficacy^15^. This is consistent with our conclusions, although we only analyzed CEA and CA199 without consideration of CA724 and other GC markers.

In addition to the aforementioned tumor markers, we also found that platelets and nutritional markers (albumin and prealbumin) may independently predict LN involvement. In fact, studies have reported that platelet count may be a reliable biomarker of lymph node metastasis likelihood^16,17^. Not only that, platelet-derived markers such as platelet-lymphocyte ratio are also useful biomarkers for predicting LN metastasis^18^. Platelets promote cell proliferation, angiogenesis, and epithelial-mesenchymal transition, and protect tumor cells from immune system attack by interacting with tumor cells to form microthrombi^19,20^. Therefore, the correlation between platelets and LN metastasis is reasonable. Accumulating evidence suggests that tumor-associated inflammation is tumor-promoting^21^. In our study, we mainly focused on the role of a single blood marker and therefore did not consider various derived markers of systemic inflammation. Although we did not find an independent predictive role of inflammatory cells and lymphocytes on LN metastasis, platelets showed an advantage. By interacting with inflammatory cells and lymphocytes, platelets are important coordinators of inflammation as well as immune responses^22^.

Prealbumin, also known as transthyretin, has a shorter half-life than albumin, only 2–3 days, and its levels are mainly affected by liver function and inflammation^23^. Notably, we found that both albumin and prealbumin were independent predictors of LN metastasis. It has been identified that perioperative prealbumin might be useful in predicting postoperative early recurrence of lung cancer and short-term postoperative outcomes after gastrectomy^24,25^. Although there are few studies on the assessment of LN metastasis by albumin or prealbumin, the combination of prealbumin and inflammatory response index has been implicated in metastasis in GC patients^26^. A study demonstrates the predictive value of platelet-to-albumin ratio for LN metastasis^5^. Given that both platelets and albumin are independent predictors of LN metastasis, our results are consistent with this report. In our study, we did not analyze the clinical significance of the variables calculated from peripheral blood parameters, and it is not difficult to infer that the ratio of platelets to albumin or the ratio of platelets to prealbumin would have a higher predictive value for LN metastasis. In order to more effectively assess the status of LN metastasis, we performed a combined ROC analysis and found that combining CEA, CA199, platelets, albumin, and prealbumin significantly improved the predictive power for LN metastasis compared to combining CEA and CA199 alone. This inspires clinicians that low prealbumin levels before treatment can not only guide nutritional support, but also reflect the status of LN metastasis. The risk of LN metastasis can be further evaluated according to the preoperative blood index in patients with N0 who have not been proved by imaging methods.

GC patients are often accompanied by elevated coagulation markers, such as fibrinogen and D-dimer. In fact, crosstalk between coagulation and inflammation makes them mutually reinforcing and is closely related to tumor progression and prognosis^27^. Studies have shown that preoperative plasma fibrinogen is associated with LN metastasis. Although computed tomography (CT) was more valuable in predicting lymph node metastases, CT could not predict lymph node metastases in 20.6% of patients, and 53.8% of them had plasma fibrinogen above the cutoff value^28^. The predictive value of D-dimer for LN metastasis has also been reported^29^. However, multivariate logistic regression showed that coagulation related markers were not independent risk factors for LN metastasis. Our current study did not compare the predictive power between blood parameters and CT, but the combination of CT and pre-treatment blood parameters can provide evidence for evaluating LN metastasis and then formulating treatment strategies.

Nutrition-related indicators are most used to guide perioperative nutritional support and evaluate the prognosis of tumor patients^30,31^. Prealbumin is more sensitive to changes in protein-energy status than albumin, therefore more suitable for nutritional monitoring^32^. In terms of survival of patients, albumin and prealbumin are well known as prognostic factors. But few studies compare prognostic superiority of albumin and prealbumin. After our analysis, we found that prealbumin, but not albumin, was an independent risk factor for OS and DFS in GC. Among other preoperative blood parameters, tumor markers (CA199 for OS and CEA for DFS) and D-dimer were independently associated with gastric cancer prognosis, which is consistent with previous studies.

This study has some limitations. First, it is a retrospective study with a small sample size. Second, we only focus on a subset of peripheral blood indicators before treatment, such as inflammatory status through blood cells. However, other indicators of this status, such as C-reactive protein, were not included in the analysis. Similarly, among gastric cancer markers, we only analyzed CEA and CA199. Third, we only considered the individual effects of each index, and did not analyze other markers calculated by these factors, such as NLR, SII, etc. However, we performed a joint ROC analysis by multivariate logistic regression. Fourth, as an effective method for detecting lymph node metastasis, imaging methods have not been compared with those of peripheral blood in their diagnostic performance.

## Conclusions

In conclusion, preoperative prealbumin-bound tumor markers can effectively predict LN metastasis in GC. Additionally, prealbumin was found to possess prognostic value as well.

## Methods

We retrospectively enrolled 424 patients with GC who underwent radical resection in the Department of Oncology, First Affiliated Hospital of Xi’an Jiaotong University from January 2011 to December 2018. All patients had undergone their initial diagnosis and had not received preoperative chemoradiotherapy. Patients were followed up by telephone, and the last follow-up was in December 2020. All patients underwent radical surgery (open or laparoscopic), and pathological staging was assessed according to the eighth edition of the American Joint Cancer Society (AJCC) tumor-node-metastases (TNM) classification. The involvement of LN was confirmed through a postoperative pathological examination. The patient’s overall/disease-free survival was defined as the time from the date of surgery to the patient’s death/relapse or last follow-up. Pretreatment laboratory measurements include whole blood cells, tumor markers (CEA, CA199), coagulation markers (fibrinogen, D-dimer and fibrin degradation products [FDP]), albumin and prealbumin. This study was performed in line with the principles of the Declaration of Helsinki. Approval was granted by the Ethics Committee of First Affiliated Hospital of Xi’an Jiaotong University (2015-046/8150826). Informed consent was obtained from all subjects and/or their legal guardian(s).

### Statistical analysis

Normally distributed continuous variables were expressed as means and standard deviations, and then compared using the student’s t-test. Non-normally distributed continuous variables were expressed as means and interquartile ranges, and compared using the log-rank test. Categorical variables were expressed as numbers and percentages, and were compared using the Chi-square and Fisher’s exact tests. The cutoff values for laboratory parameters were obtained from ROC curves for LN metastasis. The optimal cutoff value was determined at the maximun of the Youden Index. Multivariate logistic regression analysis was utilized to identify the risk factors for LN metastasis. A combined ROC curve analysis was conducted based on the predicted probabilities acquired through multivariate logistic regression. The diagnostic efficacy of ROC curves was compared using DeLong’s test. Univariate and multivariate Cox regression analyses were employed to identify independent prognostic factors for GC. Kaplan–Meier survival curves and log-rank tests were applied to compare the survival rates between different variable groups.

Statistical analysis and plotting were conducted using IBM SPSS Statistics software (version 20.0, RRID:SCR_016479). DeLong’s test was performed using MedCalc (version 19.4.1, RRID:SCR_015044). A two-sided P-value of less than 0.05 was considered statistically significant.

## Data Availability

The datasets generated during and/or analysed during the current study are available from the corresponding author on reasonable request.
